# Brief and long co-incubation of sperm and oocytes for in vitro fertilization: a meta-analysis of randomized controlled trials

**DOI:** 10.1186/s12884-023-05490-z

**Published:** 2023-03-23

**Authors:** Yiyue Fan, Zeyu Wu, Fang Peng, Hongyao Peng, Xin Liang, Shaomi Zhu

**Affiliations:** 1 School of Medical and Life Sciences/Reproductive &Women-Children Hospital, Chengdu University of Traditional Chinese Medicine, No.1166 Liutai Avenue, Wenjiang District, Chengdu City, 611137 Sichuan Province China; 2 The Affiliated Nanchong Central Hospital of North Sichuan Medical University, Nanchong City, Sichuan Province China

**Keywords:** Brief co-incubation, Long co-incubation, IVF-ET, Reproductive outcomes, meta-analysis

## Abstract

**Background:**

There is still no consensus on the optimal time of oocyte–sperm co-incubation during in vitro fertilization and embryo transfer (IVF-ET). The aim of this meta-analysis was to compare the effects of brief (1-6 h) and long (16-24 h) gametes co-incubation time on IVF outcomes.

**Methods:**

The study protocol was registered online through PROSPERO (CRD42022337503) and PRISMA guidelines were followed in the present study. The following databases were searched from inception to May 2022 for randomized controlled trials (RCTs): PubMed, Embase, Cochrane library, Web of Science, using search terms related to IVF, gametes, time of co-incubation and reproductive outcome measure. Studies comparing outcomes of brief co-incubation to that of long co-incubation during IVF, and reporting primary outcome (live birth rate), secondary outcomes (clinical pregnancy rate; ongoing pregnancy rate; miscarriage rate; normal fertilization rate; polyspermy rate; top-quality embryo rate; implantation rate) were searched. A total of 11 studies were included in the meta-analysis. Combined odds ratio (OR) and 95% confidence interval (CI) were calculated for the data. Statistical heterogeneity analysis between studies was assessed by Cochran Q and I^2^ statistic with a significant threshold of *P* < 0.05. Methodologic quality assessment of RCTs was made for potential risk of bias with Cochrane Risk of Bias Tool.

**Results:**

Compared to long-term co-incubation, brief co-incubation had an advantage in increasing implantation rate (OR: 1.97, 95% CI: 1.52–2.57), ongoing pregnancy rate (OR: 2.18, 95% CI: 1.44–3.29) and top-quality embryo rate (OR: 1.17, 95% CI: 1.02–1.35). However, brief co-incubation of gametes had no advantages in the live-birth rate (OR: 1.09, 95% CI: 0.72–1.65), miscarriage rate (OR: 1.32, 95% CI: 0.55–3.18), clinical pregnancy rate (OR: 1.36, 95% CI: 0.99–1.87) and polyspermy rate (OR: 0.80, 95% CI: 0.48–1.33) than long-term co-incubation. Additionally, the brief co-incubation was associated with lower normal fertilization rate (OR: 0.89, 95% CI: 0.80–0.99), compared with long co-incubation.

**Conclusions:**

Brief co-incubation of gametes had the advantages in increasing implantation rate, ongoing pregnancy rate and top-quality embryo rate than long-term co-incubation. However, the live-birth rate displayed no difference between the two in vitro fertilization methods. Gametes co-incubation time should be individualized according to each patient’s IVF history, infertility causes and the semen parameters.

## Introduction

IVF-ET is now widely used to treat infertile couples. During standard IVF, oocytes and spermatozoa are usually incubated for 16–24 h and fertilization. Several studies have reported the potential harmful effects of long co-incubation on oocytes and sperm [1–3]. Some scholars found prolong co-incubation may produce high levels of reactive oxygen species (ROS) which can affect the interaction of gametes and the quality of embryos [4, 5]. Exposure to ROS not only lead to hardening and thickness of the zona pellucida but also impair the implantation capacity of embryos [6]. Moreover, ROS is one of the main factors of DNA strand breaks in sperm [7]. Though DNA damaged sperm are able to fertilize oocytes, high rates of DNA breaks can have a negative impact on fertility [8, 9]. Gianaroli et al. indicated the sperm-oocyte interaction occurred when exposed to sperm within 1 h. Meanwhile, the spermatozoa entered the cumulus cells within 15 min and first appeared in the oocyte cortex after a 4 h co-incubation [10]. These studies suggested that prolonged co-incubation of oocytes and sperm might be unnecessary and even be harmful. In order to avoid the possible harmful effects on oocytes and embryos exposure to sperm at a long-term, the brief incubation of gametes has been used. Some studies demonstrated that sperm and oocyte co-incubation time of 1-6 h improved IVF outcomes than long-term co-incubation [3, 10, 11]. A meta-analysis which included eight trials involving 733 women showed higher clinical and ongoing pregnancy rates following brief incubation when compared with long-term co-incubation. However, the result has been controversial because the trials involved in study were low quality and small sample sizes, and only one trial reported the live birth rate [12]. Furthermore, more and more studies found no such advantage following brief incubation when compared with long-term incubation [13, 14].

There is still no consensus on the optimal time of oocyte–sperm co-incubation during IVF. Based on the above considerations, the purpose of the present meta-analysis was to compare the effects of brief (1-6 h) and long (16-24 h) co-incubation times on IVF outcomes.

## Material and methods

### Search strategy

We searched all eligible studies in the following databases: PubMed, Embase, Cochrane library, Web of Science, using the following keywords: ‘short co-incubation, reduced co-incubation, brief co-incubation, short insemination, brief insemination, shortened culture, reduced culture, shortened exposure’ in-combination with ‘oocyte, egg, oocyte, sperm, spermatozoa, gamete’, ‘IVF, fertilization in vitro, in vitro fertilization’, and ‘live-birth rate, ongoing pregnancy rate, clinical pregnancy rate, miscarriage rate, normal fertilization rate, polyspermy rate, top-quality embryo rate, implantation rate’. RCTs published until May 2022 were included. In addition, the references of the related articles were also manually checked to obtain all potentially additional relevant studies. No attempt was made to identify unpublished studies.

### Study selection

RCTs that compared the outcomes of IVF treatments between two infertile groups: brief co-incubation group defined as oocyte co-incubated with sperms for 1–6 hours, and long co-incubation group defined as oocyte co-incubated with sperms for 16–24 hours were included without language limitations. The studies reported at least one outcome about live-birth rate; clinical pregnancy rate; ongoing pregnancy rate; miscarriage rate; normal fertilization rate; polyspermy rate; top-quality embryo rate; implantation rate.

Studies were excluded if the included subjects received an intervention other than brief co-incubation or co-incubation time less than 1 h. Studies focused on the couples with a special history of IVF, such as embryo fragmentation were also excluded. When the same authors reported two studies within 1 year, the study with smaller size was excluded to avoid double counting. Review comments, editorials, guidelines and overviews of systematic reviews were excluded. Studies were excluded if the data extraction could not be performed, and the authors of the included researches were requested to supply missing data and details by email.

The study protocol was registered online through PROSPERO (CRD42022337503) and was completed following PRISMA guidelines for systematic reviews.

### Data extraction

Two authors (Fan and Zhu) independently extracted information from each study, using the same standardized data extraction form. Disagreements were resolved through discussion with the third author (Liang). All studies were reviewed and the following characteristics were recorded: author, year of publication, design of study, study groups, maternal age, methods of co-incubation, total sample size (numbers of patients and oocytes), sperm concentration for insemination with oocytes, the primary outcome measure (live birth rate), secondary outcome (clinical pregnancy rate, ongoing pregnancy rate, miscarriage rate, normal fertilization rate, polyspermy rate, top-quality embryo rate, implantation rate).

Live birth was defined as the delivery of a live fetus after 20 weeks of pregnancy. Clinical pregnancy was defined as the discovery of a gestational sac identified by ultrasound examination. Ongoing pregnancy was defined as the existence of a gestational sac and fetal heart activity at 12 weeks, which is confirmed by ultrasound. Miscarriage was defined as the pregnancy losses up to 20 weeks gestation. The denominator of live birth rate, clinical pregnancy rate, miscarriage rate and ongoing pregnancy rate was the number of patients who had received embryo transfer. Normal fertilization rate was defined as the percentage of zygotes with two visible pronuclei among the oocytes used for co-incubation. Polyspermy rate was defined as the percentage of zygotes with more than two visible pronuclei among the oocytes used for co-incubation. Implantation rate was defined as the percentage of embryos implanted of the embryos transferred. Top-quality embryo rate was defined as the percentage of Grade I and Grade II embryos with 2 pronuclei fertilization.

When data was not clearly described in the published research, the information would be collected by contacting the corresponding author.

### Quantitative data synthesis

Extracting dichotomy data from individual research. Statistical heterogeneity analysis between studies was assessed by Cochran Q and I^2^ statistic with a significant threshold of *P* < 0.05. The fixed effect model was accepted when *P* > 0.05 and I^2^ < 50%, otherwise, the random effect model was chosen. The subgroup analysis was performed based on the method (cumulus cells removed or retained) to explore the heterogeneity. When subgroup analysis was not possible, heterogeneity was further explored by calculating the prediction interval with each study excluded in turn. The fixed or random effect model was performed to compute the combined odds ratios ratio (OR) and 95% confidence interval (CI). Forest plots were used to compare the IVF outcome of brief and long-term co-incubation times. Methodologic quality assessment of RCTs was made for potential risk of bias with the use of the Cochrane Risk of Bias Tool. The meta-analysis was conducted using RevMan 5.3 (Statacorp, USA).

## Results

### Study selection and characteristics

**Fig. 1:**
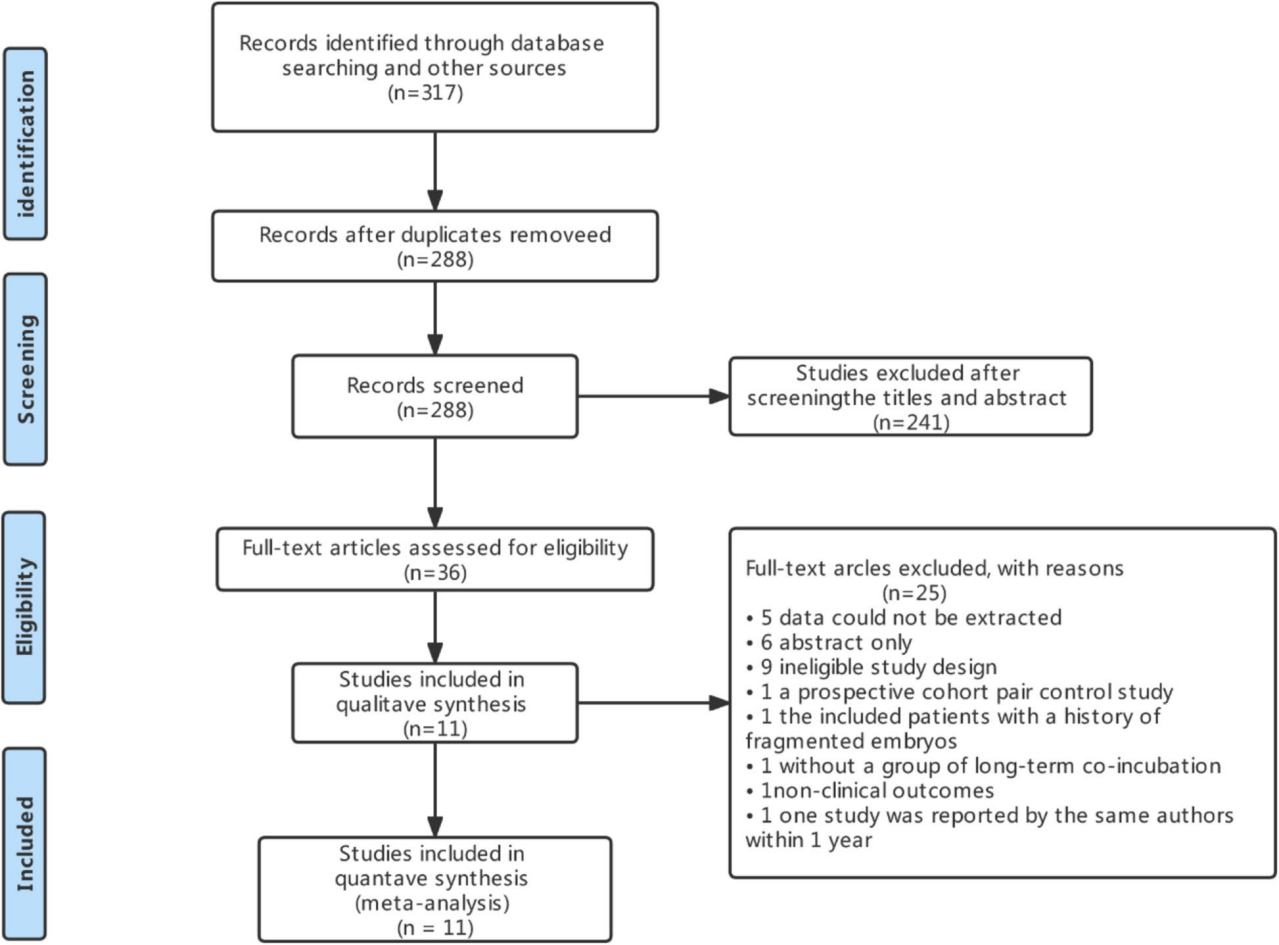
PRISMA flow chart for study identification and inclusion/exclusion

The electronic database search produced 317 potentially relevant studies. Title and abstract screening led to a total of 36 studies for full-text review; of those 25 studies were excluded. One study was reported by the same authors within 1 year. The small sample size was excluded from analysis [5]. Finally, 11 RCTs were included in the meta-analysis (Fig. 1).

We combined all studies together to calculate the total OR of the outcomes, regardless of the method of brief co-incubation intervention. One study (Lin et al., 2000) included the use of 1 h co-incubation, 3 h co-incubation, and 16–18 h long-term co-incubation [15]. To compare the brief and long-term co-incubation, we considered 1 h co-incubation and 3 h co-incubation as brief co-incubation. The Inclusion and exclusion criteria, characteristics and outcomes of included studies were summarized (Table 1 and Table 2).

**Table 1:** Inclusion and exclusion criteria in included studies

Studies	Inclusion and exclusion criteria
Kattera et al. (2003) [11]	**Inclusion criteria:** patients with tubal factor infertility, polycystic ovary syndrome, unexplained infertility, endometriosis; unable to conceive for at least 1 year. **Exclusion criteria:** very poor responders (produced < 3 follicles); men with severe oligoasthe-noteratozoospermia (density < 5 m/mL, motility < 30% and Morphology < 5%).
Dirnfeld et al. (1999) [3]	**Inclusion criteria:** patients with tubal factor infertility, unexplained infertility, endometriosis; aged 23–41 years; uterine morphology and endometrial line were normal assessed by hysterosalpingography and ultrasound. **Exclusion criteria:** very poor responders; patients with polycystic ovary syndrome; men with severe oligozoospermia.
Swenson et al. (2000) [2]	Patients were eliminated from the study for deferred transfer related to inadequate endometrial thickness, endometrial echo pattern, or risk of ovarian hyperstimulation syndrome.
Chen et al. (2019) [16]	**Inclusion criteria:** patients with tubal factor, endometriosis, unexplained and mild male factor; aged < 43 years. **Exclusion criteria:** use of donor eggs/sperm; an abnormal uterine cavity shown on hysterosalpingogram or hysteroscopy; hydrosalpinges without treatment; endometrial thickness < 8 mm on day of hCG; severe oligospermia (total number of motile sperm < 0.1 million) or normal morphology < 5%; fertilization failure or fertilization rate < 30% for conventional insemination in the past; any risk of ovarian hyperstimulation syndrome (OHSS); blastocyst transfer; and natural cycles or mild stimulation protocols (freeze all strategy).
Pongsuthirak (2019) [17]	**Inclusion criteria:** patients with tubal disease, endometriosis, ovulatory dysfunction, unexplained infertility; aged 20–38 years; at least 6 retrieved oocytes and normal semen parameters.
Coskun et al. (1998) [18]	**Inclusion criteria:** patients with tubal disease, unexplained infertility, male factor, endometriosis, polycystic ovary syndrome. **Exclusion criteria:** patients with no fertilization in both groups.
Boone et al. (2001) [19]	**Inclusion criteria:** patients with tubal factor, ovulatory dysfunction, endometriosis, pelvic factor, uterine factor; aged 23–40 years.
Gianaroli et al. (1996) [5]	**Inclusion criteria:** patients with tubal factor, idiopathic infertility, a male factor; aged ≤38 years; uterine morphology (assessed by hysteroscopy) and endometrial biopsies were normal.
Lundqvist et al. (2001) [20]	**Inclusion criteria:** patients with tubal disease, endometriosis, anovulation, a male factor, unexplained infertility; aged 25–40 years; 1–10 years of infertility. **Exclusion criteria:** less than 6 retrieved oocytes.
Barraud-Lang et al. (2008) [13]	**Inclusion criteria:** patients with tubal disease, endometriosis, dysovulation, mild male factor, and Idiopathic. **Exclusion criteria:** less than four retrieved oocytes; fertilization failure in both groups.
Lin et al. (2000) [15]	Couples without male infertility factors were selected for this study.

**Table 2:** Characteristics of studies included in the meta-analysis

Study	Design of study	Methods of brief insemination	Study groups	Maternal age (years)	Sperm concentration	Total sample size	Outcome measure
Kattera et al. (2003) [11]	RCT	Cumulus cell removed	2 h 20 h	2 h:35.4 ± 4.1 20 h:35.1 ± 3.9	2 h: 20–30 × 10^3^ spermatozoa and 1 × 10^6^ spermatozoa per oocyte 20 h: 20–30 × 10^3^ spermatozoa per oocyte	2 h: total 130 patients (1105 oocytes) 20 h: 129 patients (1200 oocytes)	Normal fertilization rate Abnormal fertilization rate Top-quality embryo rate Ongoing pregnancy rate Implantation rate
Dirnfeld et al. (1999) [3]	RCT	Cumulus cell retained	1 h 16–24 h	1 h:32.8 ± 3.8 16-24 h:33.2 ± 4.2	Both 20–50 × 10^3^ per oocyte	1 h: 72 patients (732 oocytes) 16–24 h: 86 patients (822 oocytes)	Top-quality embryo rate Implantation rate Pregnancy rate
Swenson et al. (2000) [2]	RCT	Cumulus cell removed	2 h overnight	The groups were comparable in terms of age	Both 25 × 10^3^ per oocyte	2 h: 31 patients overnight: 35 patients	Clinical pregnancy rate
Chen et al. (2019) [16]	RCT	Cumulus cell retained	3–4 h 20 h	3–4 h: 31.7 ± 4.3 20 h: 31.9 ± 3.9	Both 20–30 × 10^3^ per oocyte	3–4 h: 128patients 20 h: 144patients	Live-birth rate Ongoing pregnancy rate Clinical pregnancy rate Miscarriage rate Implantation rate
Pongsuthirak (2019) [17]	RCT	Cumulus cell retained	4 h 16-18 h	32.4 ± 2.3 (20–38)	Both 15–50 × 10^3^ per oocyte	4 h: 32patients (352 oocytes) 16–18 h: 28patients (363 oocytes)	Live-birth rate Ongoing pregnancy rate Clinical pregnancy rate Top-quality embryo rate Normal fertilization rate polyspermy rate Implantation rate
Coskun et al. (1998) [18]	RCT	Cumulus cell removed	1 h 18 h	32.1 ± 4.9	Both 0.7–3 × 10^6^ spermatozoa per m/L	Total 36 patients 1 h: 229 oocytes 18 h: 235 oocytes	Top-quality embryo rate
Boone et al. (2001) [19]	RCT	Cumulus cell removed	3 h 19 h	32.8 (range, 23–40)	Both20 × 10^3^ spermatozoa per m/L	Total 20 patients 3 h: 165 oocytes 19 h: 168 oocytes	Polyspermy rate
Gianaroli et al. (1996) [5]	RCT	Cumulus cell removed	1 h 16 h	1 h: 32.7 ± 3 16 h: 32 + 3.4	Both 2–10 × 103 spermatozoa per microdroplets	1 h: 85 patients (595 oocytes) 16 h: 82 patients (555 Oocytes)	Normal fertilization rate Polyspermy rate Implantation rate Clinical pregnancy rate Aborting rate Ongoing pregnancy rate Top-quality embryo rate
Lundqvist et al. (2001) [20]	RCT	Cumulus cell removed	2 h 18 h	32 (25–40)	2.5 × 10^5^ spermatozoa per dish	2 h: 26 patients (488 oocytes) 18 h:35 patients (504 oocytes)	Normal fertilization rate Polyspermy rate Top-quality embryo rate Implantation rate Live birth rate
Barraud-Lang et al. (2008) [13]	RCT	Cumulus cell removed	1 h 18 h	34.0 ± 4.2	Both1.5× 10^5^ spermatozoa per m/L	Total 40 patients 1 h: 1232 oocytes 18 h: 1315oocytes	Normal fertilization rate Polyspermy rate Top-quality embryo rate
Lin et al. (2000) [15]	RCT	Cumulus cell retained	1 h 3 h 16–18 h	N/A	Both 50–100 × 10^3^ Spermatozoa per oocyte	1 h: 8 patients (34 oocytes) 3 h: 14 patients (78 oocytes) 16–18 h: 22patients (216 oocytes)	Normal fertilization rate Abnormal fertilization rate Top-quality embryo rate

### Primary outcomes

#### Live birth rate

**Fig. 2:**
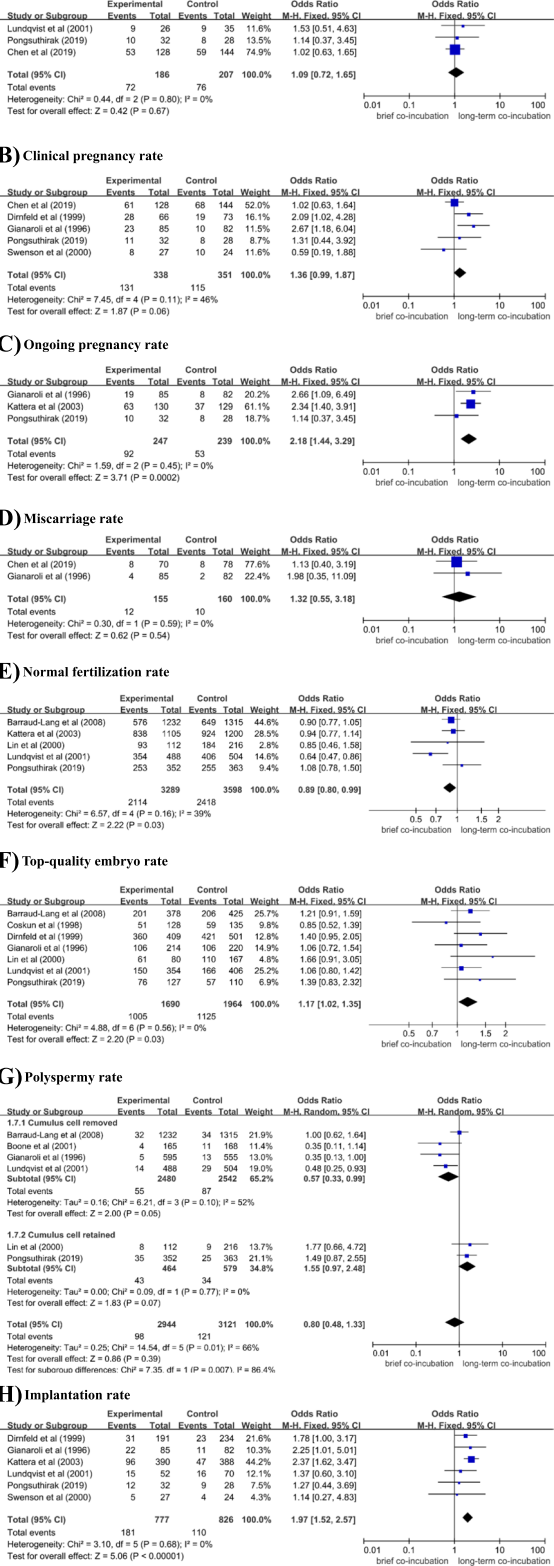
Forest plot of IVF outcomes with brief (1-6 h) or long (16-24 h) oocyte–sperm co-incubation time. Odds ratios and weighted mean differences for the outcomes: (**A**) live-birth rate; (**B**) clinical pregnancy rate; (**C**) ongoing pregnancy rate; (**D**) miscarriage rate; (**E**) normal fertilization rate; (**F**) top-quality embryo rate; (**G**) polyspermy rate; (**H**) implantation rate

Three studies included in this meta-analysis reported live birth data. There were 393 women received embryo transfer in total. 186 patients in brief co-incubation group and 207 patients in long-term co-incubation. The analysis showed there was no statistically significant difference in live birth rate (OR: 1.09, 95% CI: 0.72–1.65, Fig. 2A). There was no significant group heterogeneity (test for heterogeneity, I^2^ = 0%, *p* = 0.80, fixed effect model).

### Secondary outcomes

#### Clinical pregnancy rate

Five studies included in the meta-analysis reported clinical pregnancy data. There were 689 women in total. 338 patients in brief co-incubation group and 351 patients in long-term co-incubation. There was no statistically significant difference in clinical pregnancy rate (OR: 1.36, 95% CI: 0.99–1.87, Fig. 2B). There was no significant group heterogeneity (test for heterogeneity, I^2^ = 46%, *p* = 0.11, fixed effect model).

### Ongoing pregnancy rate

Four studies included in the meta-analysis reported ongoing pregnancy data. There were 758 women in total. 375 patients in brief co-incubation group and 383 patients in long-term co-incubation. A statistically significant increase in the ongoing pregnancy rate was observed in the brief co-incubation group when compared with long-term co-incubation (OR: 1.62, 95% CI: 0.94–2.79). There was a significant group heterogeneity (test for heterogeneity, I^2^ = 60%, *p* = 0.06, random effect model). Sensitivity analysis showed one study (Chen et al., 2019) was the source of heterogeneity. The study was removed after discussion. After removing the study, there were 486 women in total. 247 patients in brief co-incubation group and 239 patients in long-term co-incubation. There was no significant group heterogeneity (test for heterogeneity, I^2^ = 0%, *p* = 0.45, fixed effect model). A statistically significant increase in the ongoing pregnancy rate was observed in the brief co-incubation group when compared with long-term co-incubation (OR: 2.18, 95% CI: 1.44–3.29, Fig. 2C).

### Miscarriage rate

Two studies included in the meta-analysis reported Miscarriage data. There were 315 women in total. 155 patients in brief co-incubation group and 160 patients in long-term co-incubation. There was no significant difference in Miscarriage rate (OR: 1.32, 95% CI: 0.55–3.18, Fig. 2D). There was no significant group heterogeneity (test for heterogeneity, I^2^ = 0%, *p* = 0.59, fixed effect model).

### Normal fertilization rate

Six studies included in the meta-analysis reported normal fertilization data. There were 8037 oocytes used for co-incubation in total. 3884 oocytes in brief co-incubation group and 4153 oocytes in long-term co-incubation. There was no significant difference in normal fertilization rate (OR: 0.95, 95% CI: 0.78–1.14). There was a significant group heterogeneity (test for heterogeneity, I^2^ = 68%, *p* = 0.009, random effect model). Sensitivity analysis showed one study (Gianaroli et al., 1996) was the source of heterogeneity. The study was removed after discussion. After removing the study, there were 6887 oocytes used for co-incubation in total. 3289 oocytes in brief co-incubation group and 3598 oocytes in long-term co-incubation. There was no significant group heterogeneity (test for heterogeneity, I^2^ = 39%, *p* = 0.16, fixed effect model). A statistically significant decrease in the normal fertilization rate was observed in the brief co-incubation group when compared with long-term co-incubation (OR: 0.89, 95% CI: 0.80–0.99, Fig. 2E).

### Top-quality embryo rate

Seven studies included in the meta-analysis reported data on top-quality embryo. There were 3654 oocytes used for co-incubation in total. 1690 oocytes in brief co-incubation group and 1964 oocytes in long-term co-incubation. A statistically significant increase in the top-quality embryo rate was observed in the brief co-incubation group when compared with long-term co-incubation (OR: 1.17, 95% CI: 1.02–1.35, Fig. 2F). There was no significant group heterogeneity (test for heterogeneity, I^2^ = 0%, *p* = 0.56, fixed effect model).

### Polyspermy rate

Six studies included in the meta-analysis reported polyspermy data. There were 6065 2PN zygotes cleaved in total. 2944 embryos in brief co-incubation group and 3121 embryos in long-term co-incubation. There was no significant difference in polyspermy rate (OR: 0.80, 95% CI: 0.48–1.33). There was a significant group heterogeneity (test for heterogeneity, I^2^ = 66%, *p* = 0.01, random effect model). A subgroup analysis was then performed based on the method of brief co-incubation (cumulus cells removed or retained). There was no significant difference in polyspermy rate in the subgroup of removed cumulus cells (OR: 0.57, 95% CI: 0.33–0.99). There was no significant difference in polyspermy rate in the cumulus cell retained subgroup (OR: 1.55, 95% CI: 0.97–2.48, Fig. 2G).

### Implantation rate

Seven studies included in the meta-analysis reported implantation data. There were 2113 embryos transferred in total. 1015 embryos in brief co-incubation group and 1098 embryos in long-term co-incubation. A statistically significant increase in the implantation rate was observed in the brief co-incubation group (OR: 1.56, 95% CI: 1.10–2.23). There was a significant group heterogeneity (test for heterogeneity, I^2^ = 52%, *p* = 0.05, random effect model). Sensitivity analysis showed one study (Chen et al., 2018) was the source of heterogeneity. The study was removed after discussion. After removing the study, there were 1603 embryos transferred in total. 777 embryos in brief co-incubation group and 826 embryos in long-term co-incubation. There was no significant group heterogeneity (test for heterogeneity, I^2^ = 0%, *p* = 0.68, fixed effect model). A statistically significant increase in the implantation rate was observed in the brief co-incubation group when compared with long-term co-incubation (OR: 1.97, 95% CI: 1.52–2.57, Fig. 2H).

### Risk of bias

**Fig. 3:**
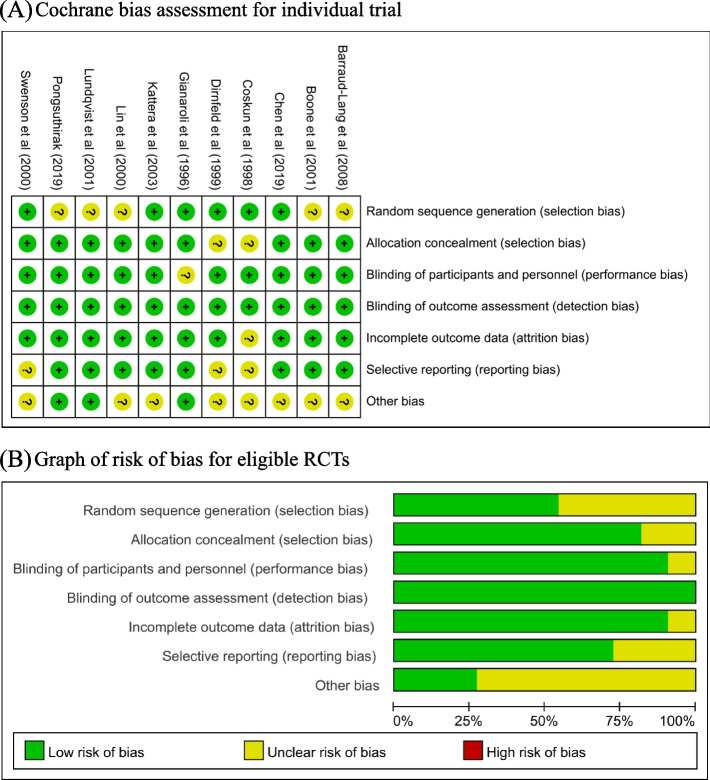
Bias analysis of the including researches. **A** Cochrane bias assessment for individual trial. **B** Graph of risk of bias for eligible RCTs

Risk of bias assessments is shown in Fig. 3 for included RCTs.

## Discussion

In the present meta-analysis, 11 studies evaluating 1183 women to compare the effects of brief (1-6 h) and long (16–24 h) co-incubation times on IVF outcome were included. This study demonstrated that brief co-incubation was associated with the increase of implantation rate (OR: 1.97, 95% CI: 1.52–2.57), ongoing pregnancy rate (OR: 2.18, 95% CI: 1.44–3.29) and top-quality embryo rate (OR: 1.17, 95% CI: 1.02–1.35) when compared with long-term co-incubation. However, the study found no advantage of brief co-incubation in live-birth rate (OR: 1.09, 95% CI: 0.72–1.65) and clinical pregnancy rate (OR: 1.36, 95% CI: 0.99–1.87) than long-term co-incubation. The result is contrary to a previous meta-analysis, which reported a significant increase of the clinical pregnancy rate in brief co-incubation group than long-term co-incubation group [21]. However, in Zhang et al. ‘s research, we found only two studies reported the clinical pregnancy rate and the two studies had a moderate risk of bias. Thus, given the limitations in sample size and the quality of studies, the reliability of the outcomes should be treated cautiously. The present study demonstrated that brief co-incubation had no advantage in decreasing the rate of polyspermy (OR: 0.80, 95% CI: 0.48–1.33, I^2^ = 66%, *p* = 0.01, random effect model), compared with long-term co-incubation. We performed a subgroup analysis for brief co-incubation methods (cumulus cells removed or retained). The result showed there was no significant difference in polyspermy rate in the subgroup of removing cumulus cell (OR: 0.57, 95% CI: 0.33–0.99) and retaining cumulus cell (OR: 1.55, 95% CI: 0.97–2.48). Additionally, the present study found brief co-incubation was associated with the decrease of normal fertilization rate (OR: 0.89, 95% CI: 0.80–0.99), compared with long-term co-incubation.

Previous study suggested brief co-incubation may decrease the levels of ROS, causing a decrease in DNA fragmentation and an increase in membrane fluidity [22]. Moreover, Dirnfeld et al. determined a short exposure of oocytes to sperm had the positive effect on zona pellucida thickness and embryo quality [6]. However, some studies found a statistically significant decrease in the normal fertilization rate in the brief co-incubation group when compared with long-term co-incubation group [11, 13, 15, 20]. Our study found a statistically significant decrease in the normal fertilization rate in the brief co-incubation group than long-term co-incubation group (OR: 0.89, 95% CI: 0.80–0.99). The possible reason was given that, oocyte denudation was performed more than 10 hours later in long (16–24 h) co-incubation than in brief (1-6 h) co-incubation after HCG trigger. Longer in vitro culture time in long co-incubation increased the chance of immature oocytes developing into mature oocyte, which might increase normal fertilization rate. We consider brief interval co-incubation may not allow adequate time for oocyte maturation of the cohort compared to longer co-incubation times. And brief co-incubation (1-6 h) could require embryology intervention in the late afternoon or in the evening which results in a longer workday for the embryology staff. Those consideration may also limit the clinical relevance and applicability of brief co-incubation.

An important factor affecting the results of IVF is whether the cumulus cells of oocytes are retained and removed after brief co-incubation. The granulosa cells in the cumulus-oocyte complex (COC) may contribute to ROS production [1]. Cumulus clouds and coronal cells could release estradiol, which may have toxic effects on embryos [23]. The most obvious advantage of cumulus cell removal was that rescue ICSI could be performed on oocytes whose second polar body was not obvious after 6 hours of co-incubation. Brief co-incubation of gametes combined with early rescue ICSI was a great option for complete fertilization failure after IVF, and the clinical results were negatively correlated with rescue time interval [24]. Emery et al. reported that embryos from delayed ICSI after in vitro maturation had increased aneuploidy, which may be caused by abnormal nuclear maturation [25]. Furthermore, the clinical pregnancy rate was 51.43% in cycles receiving rescue ICSI after 6 h co-incubation of gametes, while no clinical pregnancy was obtained in cycles receiving rescue ICSI after 20 h co-incubation of gametes [26]. However, the mechanical stress from the denuding pipette may adversely affect the zygote in the process of removing cumulus cells because the zygote is especially vulnerable soon after fertilization [27]. Another disadvantage of removal of COC could be an interfere with the important communication between the oocyte and the COC. Partial cumulus removal before insemination could decrease the normal fertilization rate and Day 3 embryo quality in humans [28].

Patients with advanced parturient age or experienced repeated IVF cycles may be susceptible to ROS and patients with abnormal spermatozoa may produce more ROS [29]. However, the result of Chen et al’ study showed age or the experience of repeated IVF cycles could not influence the outcomes of pregnancy [16]. A study focused on couples with a history of embryo fragmentation identified the subpopulation may benefit from the brief co-incubation. In addition, the study suggested that the women’s age influenced the rate of fertilization [4].

### Advantages and limitations of the study

Only RCTs were included in the present meta-analysis, and retrospective and case control studies were excluded, which ensures the reliability of the results. All studies included in this meta-analysis adopted the common design of comparing a group of patients or gametes after brief co-incubation (1-6 h) with a group using long-term co-incubation (16-24 h). Furthermore, we consolidated a variety of RCTs from various countries including 1183 cases provides a comprehensive summary to compare the effects of brief and long co-incubation times on IVF outcome.

The subgroup analysis of brief co-incubations (1, 2, 3 or 4 h) failed to detect significant differences because of too small number of cases in each time group. The same problem occurs in the statistical analysis of miscarriage rate. The confidence interval of statistical analysis is wide due to few abortion cases in each group. Therefore, effect of fertilization method on miscarriage rate should be concluded carefully. The total fertilization failure rate/low fertilization rate between the two groups because these two issues are so important for brief co-incubation of sperm and oocytes for in vitro fertilization. However, we found the two indicators were not mentioned in 10 of the 11 RCTs. We only obtained data from Chen et al. that can be used for statistical analysis by contacting the corresponding authors. Thus, given the limitations in sample size, we can only give up comparing the two indicators.

## Conclusions

The present meta-analysis has demonstrated that brief co-incubation of gametes had the advantages in increasing implantation rate, ongoing pregnancy rate and top-quality embryo rate when compared with long-term co-incubation. However, the primary outcome live-birth rate displayed no difference between the two in vitro fertilization methods. Furthermore, we can’t ignore that brief co-incubation was associated with the decrease of normal fertilization rate, compared with long-term co-incubation. Though various studies have shown that brief co-incubation of gametes combined with early rescue ICSI may be a great option for complete fertilization failure after IVF, the choice of brief co-incubation should be individualized according to each patient’s IVF history, infertility causes and the semen parameters.

## Data Availability

The datasets used and/or analyzed during the present study are available from the corresponding author on reasonable request.
